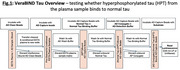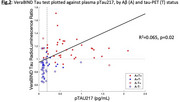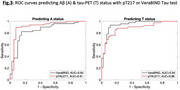# VeraBIND Tau test, a novel plasma assay for active tau pathology, identifies individuals with positive tau‐PET signal, regardless of amyloid status

**DOI:** 10.1002/alz70856_105556

**Published:** 2026-01-07

**Authors:** Bernard J Hanseeuw, Jean‐Louis Bayart, Emilien Boyer, Lisa Quenon, Pascal Kienlen‐Campard, Renaud Lhommel, Adrian Ivanoiu, Khairul Ansari, Joshua Soldo, Khalid Iqbal

**Affiliations:** ^1^ Massachusetts General Hospital, Gordon Center for Medical Imaging and the Athinoula A. Martinos Center for Biomedical Imaging, Boston, MA, USA; ^2^ Department of Neurology, Saint‐Luc University Hospital, Brussels, Belgium; ^3^ Institute of Neuroscience, UCLouvain, Brussels, Belgium; ^4^ Institute of Neuroscience, UCLouvain, Brussels, 1200, Belgium; ^5^ Departement of Laboratory Medicine Cliniques Saint Pierre, Ottignies, Belgium; ^6^ Grand Hôpital de Charleroi, Charleroi, Belgium; ^7^ Institute of Neuroscience ‐ UCLouvain, Brussels, Belgium; ^8^ Saint‐Luc University Hospital, Brussels, Belgium; ^9^ Veravas, Inc, Oakdale, MN, USA; ^10^ New York State Institute for Basic Research in Developmental Disabilities, Staten Island, NY, USA; ^11^ SUNY Downstate/NYSIBR Center for Developmental Neuroscience, Staten Island, NY, USA

## Abstract

**Background:**

Plasma assays targeting tau phospho‐epitopes (e.g., pTau217) are frequently observed in the context of isolated amyloid‐β pathology (A+). Developing plasma assays associated with tau aggregation (T+) is an unmet challenge. Here, we present results of the VeraBIND Tau test, a plasma assay measuring how hyperphosphorylated tau (HPT) observed in a sample binds to normal tau, i.e., testing whether tau is pathologically active.

**Method:**

VeraBIND Tau is an in vitro bead‐based ELISA that uses chemiluminescence (Figure 1: overview). One hundred thirty‐three participants were recruited from an ongoing study at UCLouvain, Belgium, including 93 clinically normal (CN, 32A+) and 40 clinically impaired (MCI/AD, 36A+). A+ status was determined using either CSF (Aβ_42_≤544pg/mL) or amyloid‐PET (Centiloid≥20). ^F18^MK6240 Tau‐PET status was determined visually as negative (Braak 0) or positive (Braak≥1). Plasma pTau217 was quantified using Lumipulse (Fujirebio).

**Result:**

The VeraBIND Tau plasma assay was weakly correlated with pTau217 concentration (R^2^=0.065, *p* = 0.02, Figure 2). Using tau‐PET as ground truth for tau, VeraBIND Tau detected 52 of 56 A+T+ participants (sensitivity=93%), and excluded tauopathy in 54 of 57 A‐T‐ participants (specificity=95%). With the same specificity (threshold=0.193pg/mL), pTau217 achieved similar sensitivity in A+T+ (51 of 56, 91%). However, nine of twelve A+T‐ participants had positive pTau217 whereas only four had positive VeraBIND test. In contrast, six of eight A‐T+ participants had negative pTau217, but positive VeraBIND test, including one FTLD and two probable PART cases. Except the FTLD case, the A‐T+ participants had relatively low tau‐PET signal (Braak 2‐3) and the only A‐ Braak=1 case was borderline (RLU=0.974, normal<1.0). The overall diagnostic accuracy of pTau217 was 90% for A‐/A+ and 82% for T‐/T+ while the accuracy of VeraBIND was 84% for A‐/A+ and 90% for T‐/T+.

**Conclusion:**

Whereas pTau217 is more closely associated with amyloid than with tau‐PET results, VeraBIND Tau is highly sensitive to (early) tau‐PET positivity, indicating active tau aggregation, with or without amyloidosis. The mismatch between pTau217 and VeraBIND results provides a plasma indication of discordant A/T PET‐status.